# Combined latissimus dorsi transfer and deltoid flap for irreparable rotator cuff tear: A retrospective analysis

**DOI:** 10.1051/sicotj/2023034

**Published:** 2023-12-07

**Authors:** Sami Roukoz, Samuel George, Marven Aoun, Mohammad Daher

**Affiliations:** 1 Hotel Dieu de France, Orthopedics Department Beirut Lebanon

**Keywords:** Irreparable rotator cuff tears, Deltoid flap, Latissimus dorsi transfer, Double transfer, Posterosuperior rotator cuff tear

## Abstract

*Purpose*: This study will evaluate the outcomes of the combined latissimus dorsi tendon transfer and deltoid flap for the management of irreparable posterosuperior rotator cuff tears. *Methods*: This is a retrospective study of 15 patients who have undergone double transfer surgery for their irreparable posterosuperior rotator cuff tears. These patients were followed up in three periods. Functional outcomes such as the constant score (CS), subjective shoulder value (SSV), and range of motion were assessed as well as radiographic outcomes such as the acromio–humeral distance. *Results*: Fifteen patients were included in the early and intermediate follow-up while only 8 remained at the latest follow-up. At the early follow-up the SSV (*p* = 0.001), CS (*p* = 0.021), and A–H distance (*p* = 0.008) showed a statistically significant improvement from their pre-operative values. At the latest follow-up, only the external rotation decreased from its value at the intermediate follow-up (*p* = 0.027). *Conclusion*: The double transfer technique showed sustainable improvement of the functional outcomes except for the external rotation. However, the loss of external rotation did not affect the remaining outcomes. Trials comparing this technique to other surgical managements or MIRCTs are needed to confirm these results.

## Introduction

The management of massive and/or irreparable rotator cuff tear (RCT) remains a challenge. Many methods have been proposed, such as rehabilitation, arthroscopic debridement, a subacromial balloon, transfers, or a reverse shoulder arthroplasty at a later stage [1, 2]. Tendon transfers and flaps can be beneficial, especially in the younger more demanding patients. In fact, one of these options is a repair of the rotator cuff using a local flap of the deltoid from the anterior part of the acromion which was shown to report good clinical outcomes initially [3]. However, the results of the technique vary widely in the literature with some authors describing excellent results [1, 4]. Furthermore, the results of this technique were shown to decline over time [4, 5]. In addition, this procedure is technically demanding, and the deltoid flap does not prevent long-term upward migration of the humeral head which may explain in part the controversial results reported in the literature with this technique [6].

Another option is the latissimus dorsi transfer (LDT) which is a viable option for the treatment of posterosuperior RCT [7]. However, good results were not reproducible for all the authors with varied tendon insertion positions on the humeral heads being described [4, 8, 9]. In fact, the LDT can function to prevent the upward migration of the humeral head. Nevertheless, it needs a wide dissection and liberation in order to reach the anterior cuff which will jeopardize its strength. Another disadvantage of this technique is its failure to withstand the proof of time [4].

Therefore, we developed a surgical technique that combines the two approaches and concepts. This technique allows the correct cover of the humeral head by the deltoid flap associated with a modified LDT to act as a stopper to its upward migration. This technique aims to enhance the long-term clinical outcome in patients suffering from irreparable RCTs, especially in the younger demanding population. The purpose of this study was to evaluate the clinical and radiographic outcome of the double transfer technique for the treatment of posterosuperior irreparable RCTs in both the short and long term.

## Material and methods

### Patients

Between 2013 and 2017, the senior author performed on 15 patients (9 males and 6 females) 15 double transfer techniques using simultaneously the modified latissimus dorsi and the deltoid flap for the treatment of irreparable RCTs. This study was approved by the local ethics committee. The inclusion criteria were a symptomatic shoulder (painful shoulder and/or reduced range of motion) with a retracted and irreparable posterosuperior RCT as shown on ultrasound or magnetic resonance imaging. Furthermore, all tears were reclassified intra-operatively. It was determined to be irreparable if all attempts to mobilize the rotator cuff tendons were deemed to failure and we were unable to bring the tendons to the greater tuberosity with the arm at maximum of 30° of abduction. The exclusion criteria were a non-reparable subscapularis tear and anterosuperior RCT. The average age at the time of operation was 64.3 years (range: 46–82 years). All patients had a complete irreparable tear of the supraspinatus and infraspinatus with an intact deltoid. Three patients had concomitant rupture of the subscapularis that were amenable to reinsertion. Two patients had a failed rotator cuff repair and two had hemiarthroplasty for proximal humeral fractures.

**Figure 1:**
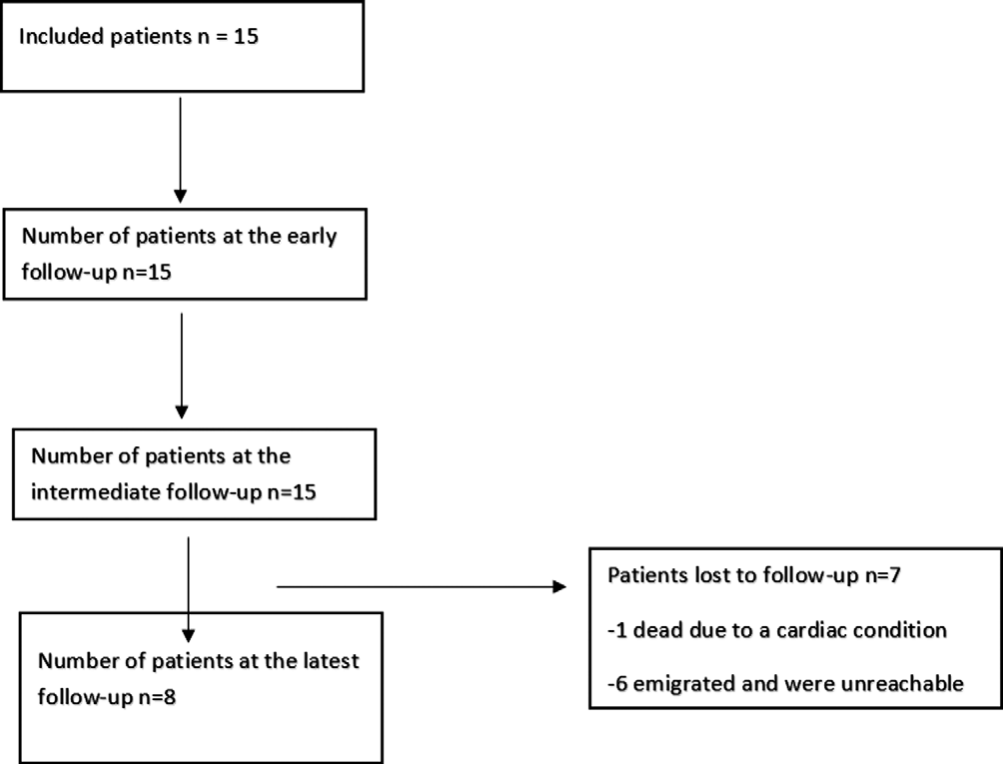
Flowchart of the included patients throughout the different stages of follow-up.

At the long-term follow-up, only eight patients were included in the last follow-up since most of the patients emigrated outside of the country and were unreachable due to the huge economic crisis (Figure 1). Furthermore, the latter did not allow us to perform standard shoulder radiographs for the patient. In addition, the SSV was not included in this follow-up since a lot of the patients had a painful contralateral shoulder due to osteoarthritis, rotator cuff arthropathy, and various other reasons, and they could not get the idea of an “ideal” contralateral shoulder. However, the CS was used as it was shown to correlate with the SSV [10].

### Clinical assessment and strength measurements

Patients were evaluated post-operatively at an early period before being re-evaluated at an intermediate period and their last follow-up was 2023. The assessment was done using a constant shoulder score (CS) questionnaire, the subjective shoulder value (SSV), and a visual analog scale (VAS) were also performed to assess pain. Furthermore, an anteroposterior radiograph was done pre-operatively and post-operatively at the early and intermediate follow-up to assess the sub-acromial space by measuring the acromion-to-humerus interval (AH interval) and glenohumeral arthritis.

### Surgical technique and post-operative management

#### Positioning and dissection

**Figure 2:**
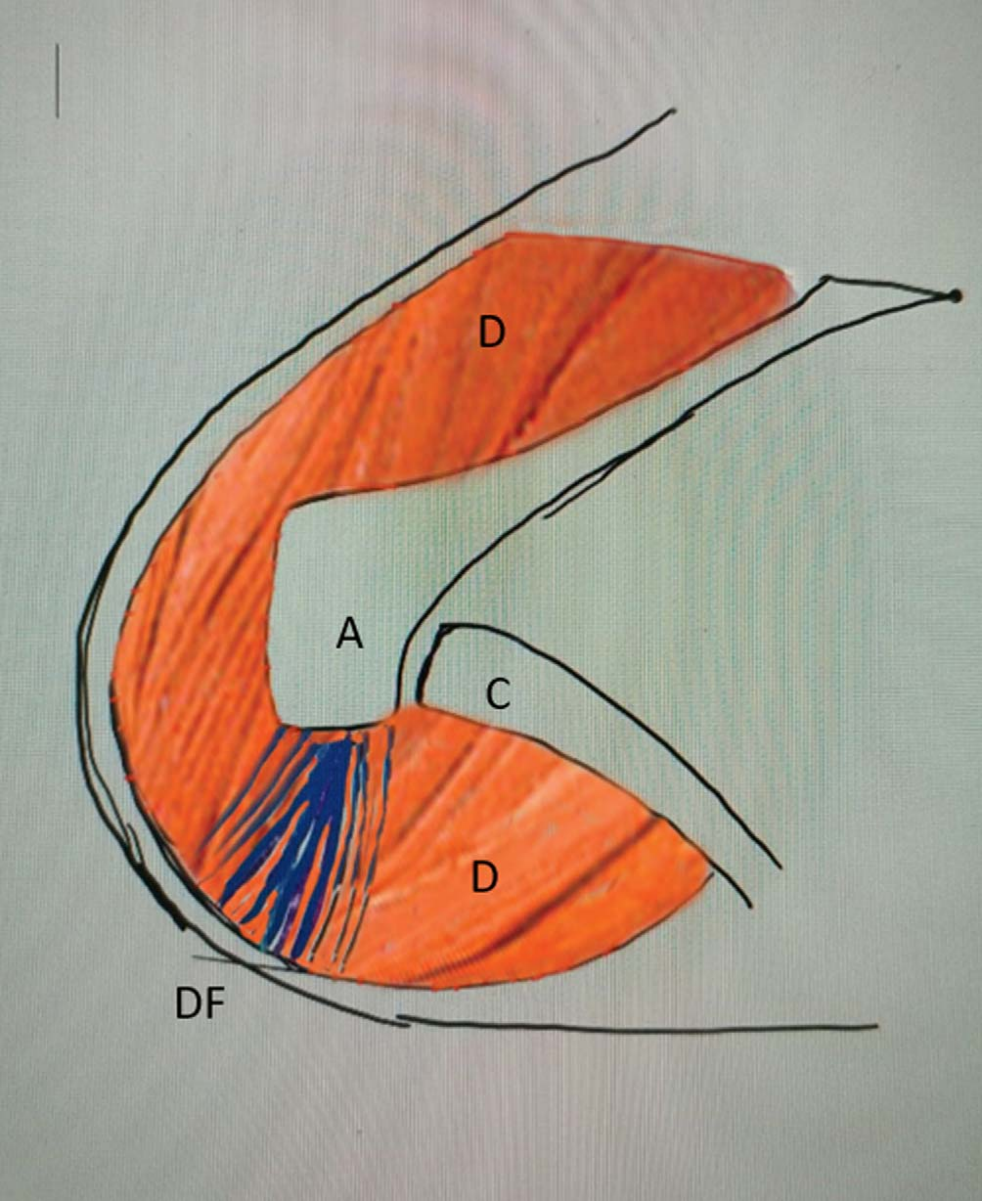
Planning of the trans-deltoid approach and deltoid flap. A – acromion, C – clavicle, D – deltoid muscle, DF – deltoid flap.

The operation was done with the patient under general or loco-regional anesthesia, in a dorsal decubitus position and a sandbag was positioned behind the affected shoulder. The rotator cuff was exposed via a 5-cm superolateral trans-deltoid split approach, with partial detachment of the origin of the posterior part of the anterior part of the middle deltoid from the acromion. The bursa was excised (Figure 2).

#### Deltoid flap

**Figure 3:**
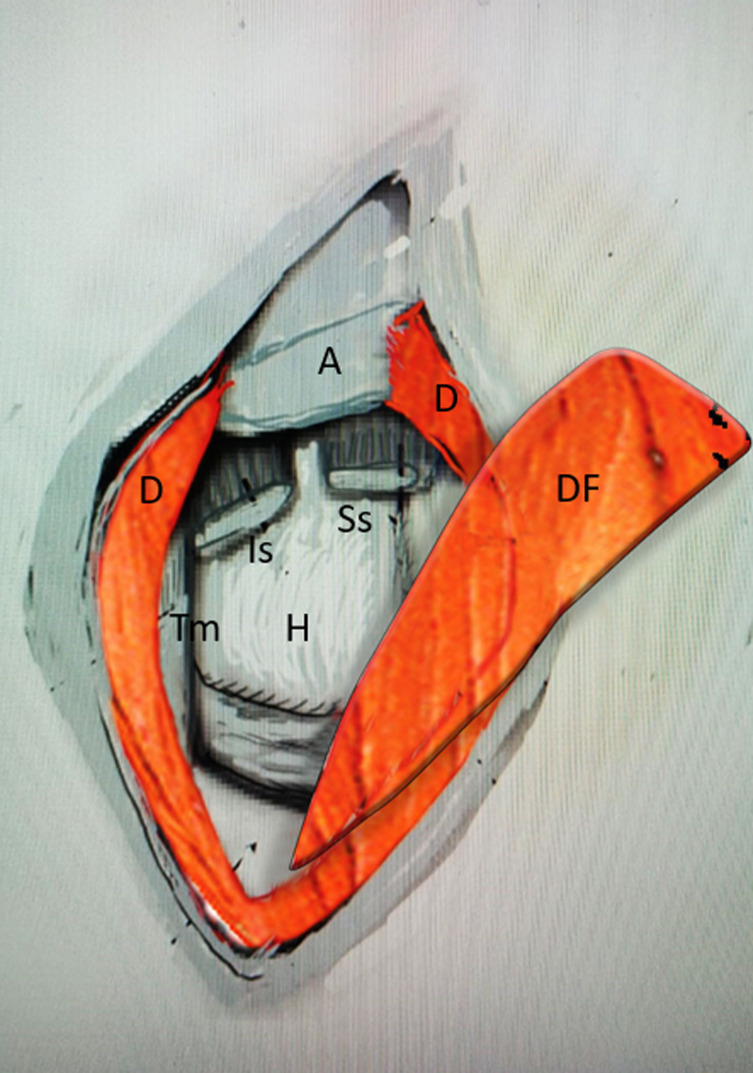
Rotator cuff debridement and elevation of the deltoid flap. A – acromion, D – deltoid muscle, DF – deltoid flap, H – humeral head, Ss – supraspinatus tendon, Is – infraspinatus tendon, Tm – teres minor.

**Figure 4:**
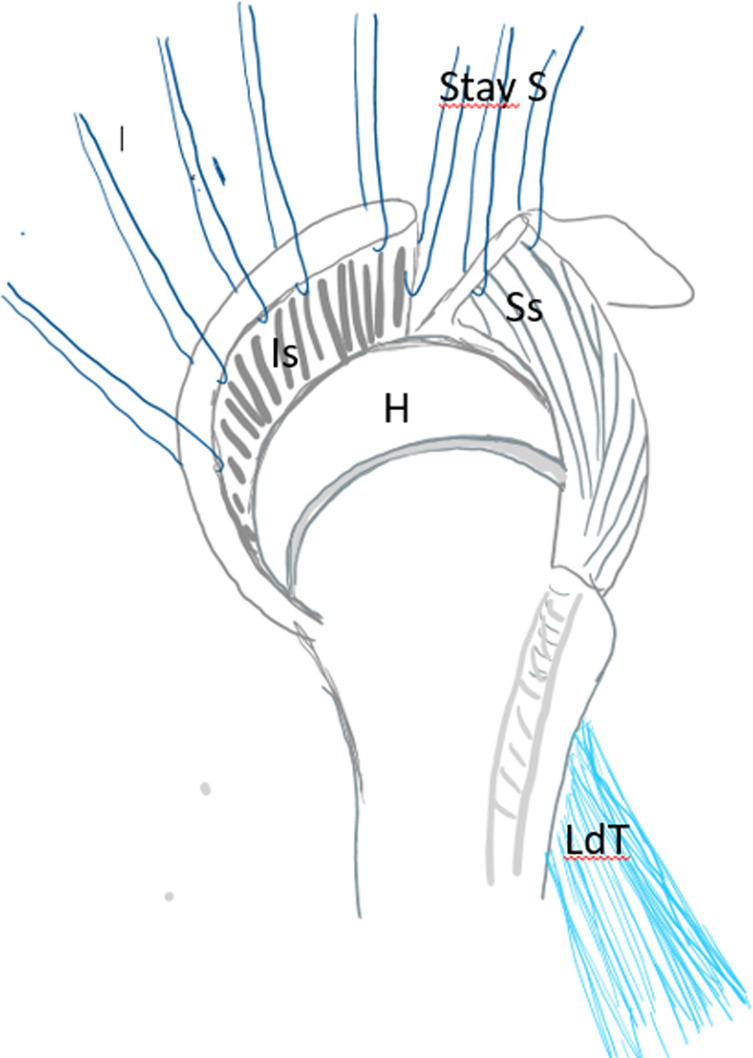
Preparing the debrided rotator cuff with stay sutures. H – humeral head, Ss – supraspinatus tendon, Is – infraspinatus tendon, LdT – latissimus dorsi tendon, Stay S – stay sutures.

After debridement of the rotator cuff, a tenotomy with a tenodesis of the long biceps at the groove as well as an acromioplasty was systematically done. Then the anterior part of the middle deltoid was separated from the anterior deltoid preserving its distal muscular connection. By doing this, the flap is elevated to be sutured later on to the debrided margin of the rotator cuff as described by Apoil et al. [11] (Figure 3). After that, many stay sutures were put on hold through the debrided cuff for later suture after transferring and reinserting the LDT (Figure 4). The subscapularis was also explored.

#### LDT

**Figure 5:**
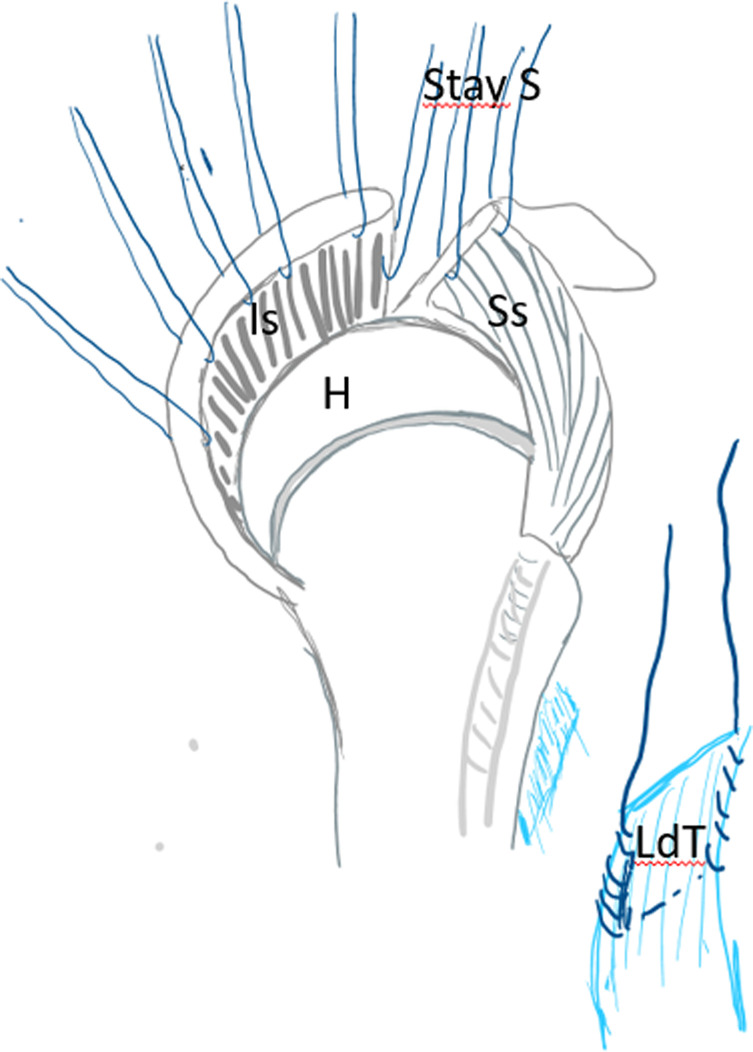
Harvesting the latissimus dorsi tendon. H – humeral head, Ss – supraspinatus tendon, Is – infraspinatus tendon, LdT – latissimus dorsi tendon, Stay S – stay sutures.

**Figure 6:**
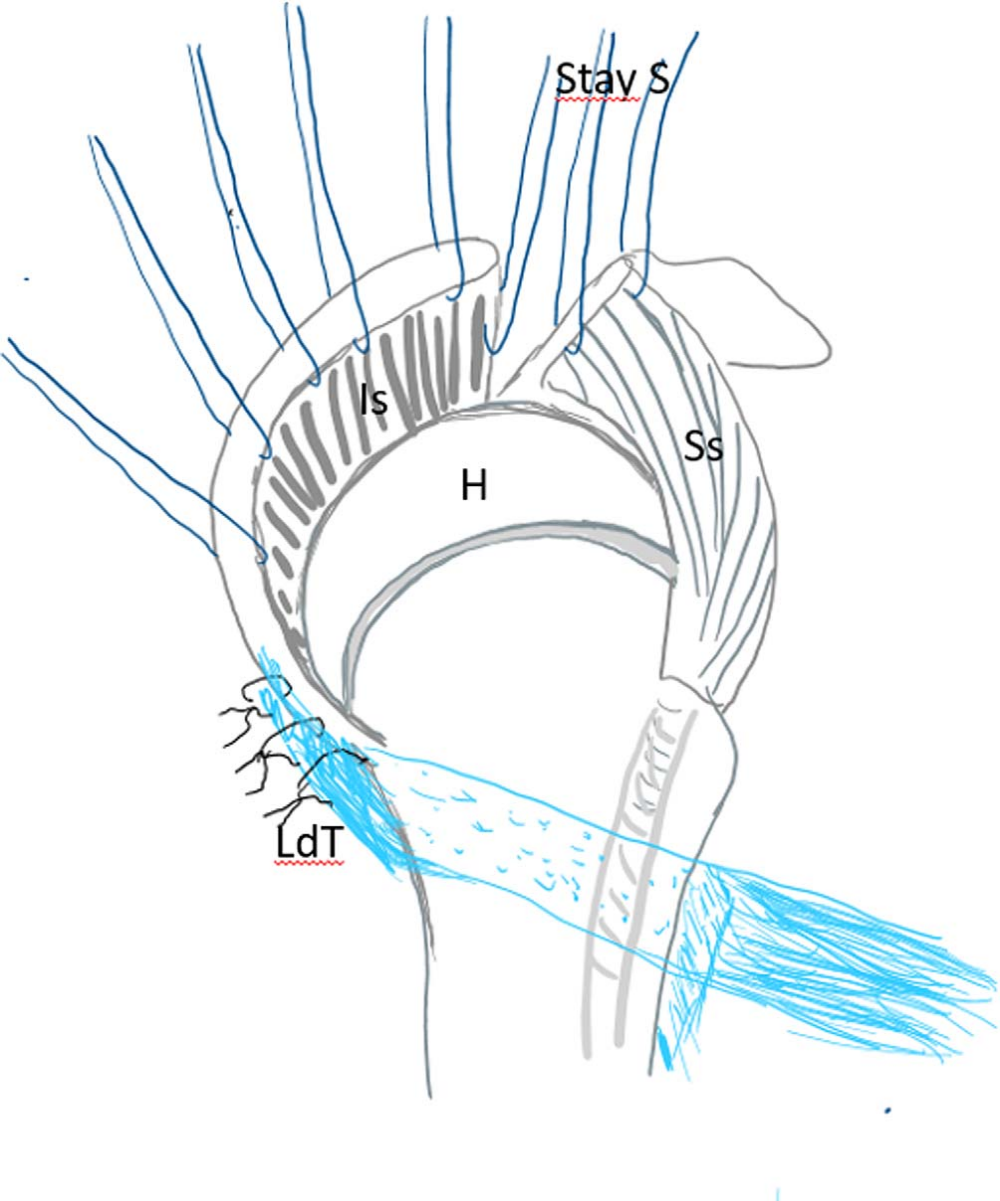
Fixing the latissimus dorsi tendon to the remnants of the infraspinatus after tunneling through the quadrangular space. H – humeral head, Ss – supraspinatus tendon, Is – infraspinatus tendon, LdT – latissimus dorsi tendon, Stay S – stay sutures.

**Figure 7:**
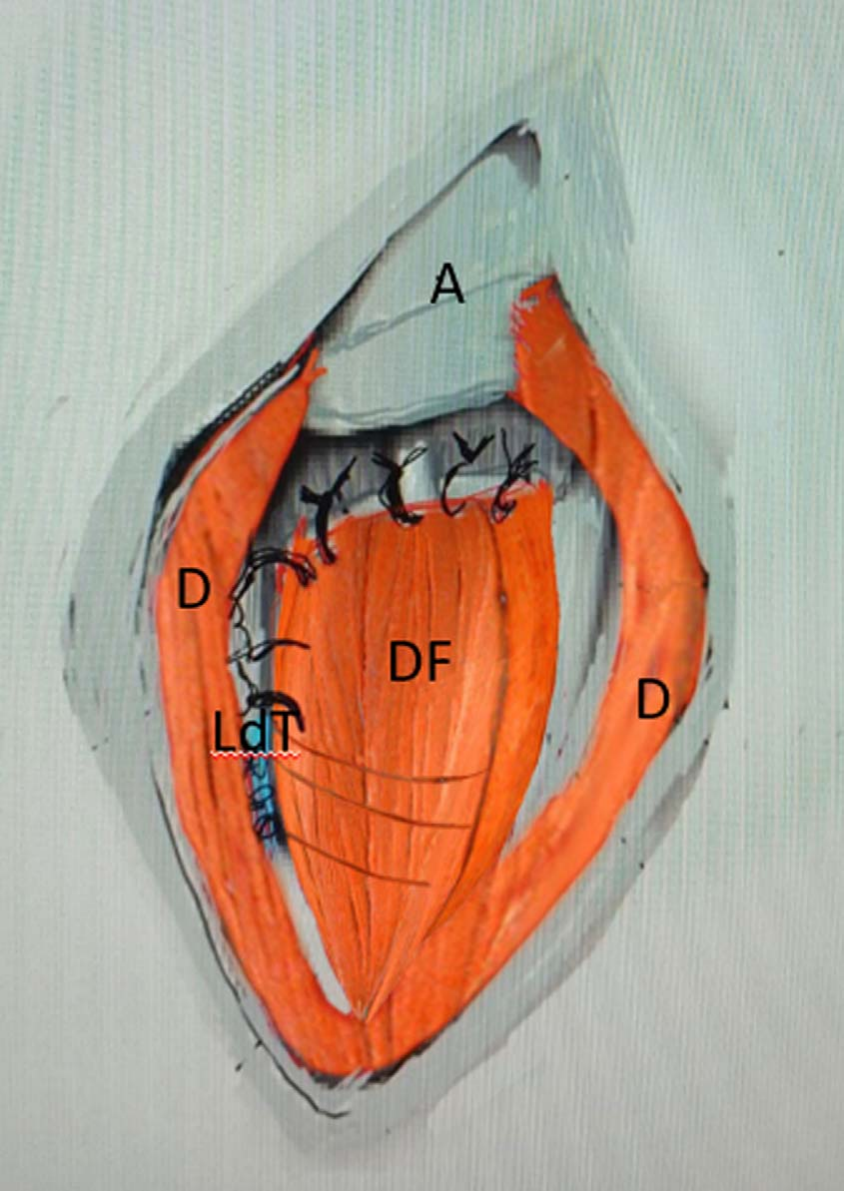
Suturing the deltoid flap to the debrided rotator cuff using the stay sutures. A – acromion, D – deltoid, LdT – latissimus dorsi tendon.

Another 5 cm incision was made along the medial proximal third of the arm. The insertion of the latissimus dorsi was identified behind the neurovascular pedicle while the arm was externally rotated and abducted by the assistant. The tendon was sharply detached from the humerus keeping the teres major intact. The neurovascular pedicle was identified and protected in the axillary crease, and only the tendinous part of the LD muscle was minimally freed from its deep fascia to allow its mobilization (Figure 5). In this way, the muscle cannot be overstretched during the transfer. A Krakow suture was made along each side of the tendon from the musculotendinous junction to the end of the tendon. A wide tunnel was made by bluntly dissecting its path and the tendon was amenable through the quadrangular space to the footprint of the infraspinatus tendon (Figure 6). By not placing the tendon anteriorly as described by most of the surgical techniques of the transfer less tension and less fatiguability was exercised on the muscle. A metallic clip was inserted on its distal part to allow its localization on imaging post-operatively. At this stage, if any residual remnants of the infraspinatus were present on the bone, the LDT was fixed to it by direct non-resorbable sutures. Otherwise, the LDT was fixed to the footprint of the infraspinatus using anchor sutures. After that, the deltoid flap was sutured to the stay sutures on the debrided rotator cuff and then the deltoid muscle was sutured to fill the gap of the harvested flap (Figure 7).

#### Postoperative management

The arm was maintained in 20°–30° of abduction in an orthosis that was kept for a total of 4 weeks. At 2 weeks postoperatively, a physiotherapy regimen was started by passive mobilization of the shoulder in external rotation, abduction, and forward elevation starting from the abduction brace position. After 4 weeks the orthosis was removed, and an arm sling was used for another 2 weeks. At 6 weeks, the strengthening phase begins with the arm at the side and then progressively with forward elevation in a supine position and then in a seated position.

### Statistical analysis

The data have been analyzed using the SPSS 25.0 software (SPSS Inc., Chicago, IL, USA). The Kolmogorov–Smirnov test was used to assess the normal distribution of the data. Since the tests came back significative, the non-parametric Wilcoxon Signed Rank test was used in order to compare the outcomes at the early follow-up to the pre-operative data, the outcomes at the intermediate follow-up to the early one, and the outcomes at the latest follow-up to the intermediate one. *p* = 0.05 was considered for the statistical significance threshold.

## Results (Table 1)

### Early and intermediate follow-up

During the early follow-up period (8.6 months), both the SSV and CS improved in a statistically significant manner (*p* = 0.001 and *p* = 0.021 respectively). A similar trend was seen in the acromio–humeral interval (*p* = 0.008). As for pain (assessed on the visual analog scale (VAS)) and range of motion, they all improved without reaching statistical significance (*p* > 0.05).

**Table 1:** Post-operative outcomes.

	Pre-operative (*n* = 15)	Early post-operative follow-up (*n* = 15)	Intermediate post-operative follow-up (*n* = 15)	Latest post-operative follow-up (*n* = 8)
Mean follow-up time (month)	–	8.6 (3.9)	43.4 (5)	96 (11.1)
Mean (SD)				
SSV (%)	18 (0.17)	64 (0.17)*	65 (16.9)**	–
Mean (SD)				
Constant (%)	37.2 (15.6)	69 (15.3)*	74 (12.6)**	84.5 (18.1)
Mean (SD)				
VAS	6.8 (1.7)	0.67 (1.5)	1 (1.9)	0.38 (1.1)
Mean (SD)				
Flexion (°)	95 (65.3)	94.4 (55.9)	108 (50.3)	151.3 (23.6)
Mean (SD)				
Abduction (°)	79 (55.9)	90 (53.56)	97 (43)	137.5 (21.9)
Mean (SD)				
External rotation (°)	16.3 (21.1)	41.25 (19.2)	50.7 (25.2)**	23.8 (7.4)***
Mean (SD)				
Acromio-humeral distance (mm)	4.7 (1.3)	9.5 (3.2)*	8.6 (2.4)**	–
Mean (SD)				

* Difference with pre-operative values is significant (*p* < 0.05).

** Difference with early post-operative follow-up values is significant (*p* < 0.05).

*** Difference with intermediate post-operative follow-up values is significant (*p* < 0.05).

When comparing the functional outcomes at the time of intermediate follow-up (43.4 months) to the previous timepoint, the SSV, CS, and external rotation were the only variables to show significant improvement (*p* < 0.0001, *p* < 0.001, and *p* = 0.011 respectively). As for the radiographic outcomes, the mean acromio–humeral interval decreased to 8.6 (*p* = 0.012). However, it remained superior to its pre-operative value.

### Long follow-up

At this follow-up (96 months), all of the assessed outcomes had no statistically significant change when compared to the intermediate follow-up except for the active external rotation which was reduced to 23.8° ± 7.4° (*p* = 0.027).

### Complications

There were no infections, permanent nerve lesions or bleeding. One patient had a pull-out of the latissimus dorsi transfer as demonstrated by the distal migration of the metallic clips in the axilla on follow-up X-rays. Despite this distal migration, the patient had good results with a constant score of 75 and SSV of 80% at the intermediate follow-up. The patient was unreachable at the latest follow-up.

## Discussion (Table 2)

The main results of our study are that at short and intermediate-term follow-up (at a mean of 8.6 and 43.4 months respectively), the SSV, CS, and external rotation all improved in a statistically significant manner. Furthermore, the A–H distance remained significantly twice the distance it was pre-operatively despite decreasing between the short and intermediate-term follow-up. At long-term follow-up (at a mean of 96 months), there was no deterioration in any of the measured outcomes except for the external rotation statistically decreasing. However, this change in range of motion did not translate into any of the functional outcome scores.

**Table 2:** Summary of the discussed studies in our manuscript.

Technique	Authors	Patients	Follow-up period	Results	Conclusion
Deltoid flap	Lu et al. [1]	18 patients (15 males and 3 females)	13.9 years	23-point increase in constant score and improvement in pain score (80%) 50% of deltoid flaps rupture and muscular strength deteriorates with time	Significant analgesic effect of deltoid transfers This technique is not recommended for further use in the management of massive, irreparable rotator cuff tears
		Mean age: 52.3 years			
	Roukoz et al. [5]	17 patients (9 males and 8 females)	3.4 years	38-point increase in constant score, 80% improvement in constant pain score and 89% improvement in patient satisfaction	Deltoid muscle flap is reported to be a good option in terms of pain relief and functional improvement
		Mean age: 61.3 years			
	Glanzmann et al. [12]	33 patients (20 males and 11 females)	4.4 and 14.6 years	25-point increase in constant score and 64% satisfaction at midterm follow-up with maintenance of these results at long-term follow-up 87% of deltoid flaps rupture at latest follow-up with progression of humeral head migration	This technique is no longer recommended for management of massive irreparable rotator cuff tears
		Mean age: 65 years			
Latissimus dorsi transfer	Gerber et al. [13]	14 patients (13 males and 1 female)	1.2 years	Patients showed an improvement in pain and range of motion	This procedure yielded promising early subjective and objective outcomes
		Mean age: 59 years			
	Clavert et al. [14]	14 patients (10 males and 4 females)	4.7 years	There was significant electrical activity in the transfer during abduction and external rotation The mean constant score increased by 22 points, the range of motion was improved, and 12 patients were satisfied Only 2 out of 14 transfers failed at the latest follow-up	Latissimus dorsi transfer is a good procedure for massive irreparable rotator cuff tears It acts not only as a muscle tenodesis that covers the humeral head, but also as an active muscle transfer
		Mean age: 52 years			
	Waltenspül et al. [15]	31 patients (23 males and 8 females)	3.5 years	The mean constant score increased by 13 points, and the subjective shoulder value increased by 34 points There was no statistically significant improvement in external rotation and abduction 13% of the transfers failed at the final follow-up	Midterm results of latissimus dorsi transfers yielded good subjective and objective improvements High rate of failure in this cohort Long-term studies are needed to assess the utility of this technique
		Mean age: 55.5 years			
	Gerber et al. [16]	55 patients (38 males and 17 females)	12.3 years	Constant score had a 24 points improvement and subjective shoulder value had 41 points improvement at the latest follow-up Range of motion improved in a statistically significant manner Only 1 traumatic failure occurred at the latest follow-up	Latissimus dorsi tendon transfer offered an effective treatment for irreparable posterosuperior rotator cuff tears, with durable improvements in shoulder function and pain relief
		57 shoulders			
		Mean age: 56 years			
	Talbot et al. [9]	16 studies	–	31% of articles reported a satisfaction rate less than 80% 67% of articles reported constant scores below 70	Results of latissimus dorsi transfer for massive irreparable rotator cuff tears are variable

The findings of this study should be taken into consideration several limitations. First, it is a retrospective study. Second, the number of patients enrolled is relatively small especially, in the latest follow-up period. Third, we did not compare the group of patients undergoing the double transfer technique to a control group. However, this study has its strengths such as following patients to a mean of 96 months post-operatively and assessing them at different time-point throughout their post-operative course.

The transfer of the deltoid flap a surgical treatment of massive RCTs was abandoned for several reasons [12]. First of all, the results at short and midterm were variables between studies [1, 5, 12]. In fact, Glanzmann et al. reported no benefits of this procedure in terms of range of motion, but only in pain and constant score [12]. As for Roukoz et al., they reported major improvement in all the subcategories of the constant score including pain, quality of life, and range of motion, except for the force section [5]. Lu et al. reported significant improvement in range of motion, quality of life, and pain. However, the improvement in the range of motion did not reach statistical significance [1]. Second, failure of the flap and subsequent upward migration of the humeral head was not shown to be a rare complication of this surgical management [1, 12]. Glanzmann et al. reported a 19% survival rate of the flap at midterm follow-up and 13% at long-term follow-up [12]. In addition, Lu et al. reported a 50% failure rate at long-term follow-up [1]. On the other hand, a systematic review conducted by Koositra et al. in 2019, including 63 studies to assess outcomes for all reported types of treatment modalities for irreparable posterosuperior RCTs showed that treatment using deltoid flap had the highest mean weighted improvement in constant score (44.1 points) with available medium- and long-term (4–5 years) follow-up [4]. However, there was a drop of improvements in constant score at long-term follow-up as shown in other management techniques such as tendon transfer and debridement

The LDT was first proposed by Gerber in 1988 for irreparable posterior RCT [13]. The LDT has shown variable results as well [9]. In fact, in a review of 16 articles, Talbot et al. found that LDT transfer for posterosuperior irreparable yielded a satisfaction rate of less than 80% in 4 out of 13 studies reporting satisfaction rates [9]. Furthermore, 8 out of 12 articles on LDT transfer were unable to reproduce a constant score above 70 with a follow-up of 50 months or less [9]. On the other hand, significant improvements in range of motion and strength are demonstrated with this surgical technique with a lower rate of failure and subsequent migration of the proximal humerus [8, 13–16]. Nevertheless, as reported above, a drop in improvement is seen at long-term follow-up [4].

The rationale of our double transfer technique is to restore the anatomy as well as the biomechanics of the rotator cuff. The deltoid flap addresses the functional deficiency of the supraspinatus as well as the anatomy of the superior capsule acting as a tendon-sealing effect over the humeral head. The LDT tries to prevent the upward migration of the humeral head as well as to restore the active range of motion and strength of the shoulder and aids the deltoid flap preventing its overcharge and thus avoiding its failure. In our technique, there was no decrease in the functional outcomes between the three follow-up periods with the last follow-up having a mean of 96 months making these results sustainable in the long term. As for radiographic outcomes, at the intermediate follow-up, the A–H distance decreased when compared to the earlier follow-up but it remained twice the distance it was pre-operatively.

## Conclusion

The simultaneous deltoid and latissimus dorsi transfer can be proposed to reconstruct deficient and irreparable posterosuperior RCT. Sustainable improvements in the functional outcomes were achieved with this technique. As for the range of motion, the latter did not deteriorate except for the external rotation which decreased at the latest follow-up. However, it did not affect the constant score. The radiographic A–H distance was improved at the early follow-up but decreased as well at the intermediate follow-up without affecting any of the functional outcomes which significantly increased at this same follow-up. Nonetheless, a trial comparing this technique to other techniques with a bigger sample of patients is needed to reach more robust results.
